# Ureteroarterial Fistula

**DOI:** 10.1155/2009/326969

**Published:** 2009-11-15

**Authors:** D. H. Kim, A. Mahdy, V. Mundra, M. Berman, G. M. Ghoniem

**Affiliations:** ^1^Department of Urology, Cleveland Clinic Florida, 2950 Cleveland Clinic Blvd. Weston, FL 33331, USA; ^2^Department of Internal Medicine, Cleveland Clinic Florida, 2950 Cleveland Clinic Blvd. Weston, FL 33331, USA; ^3^Department of Radiology, Cleveland Clinic Florida, 2950 Cleveland Clinic Blvd. Weston, FL 33331, USA

## Abstract

Ureteral-iliac artery fistula (UIAF) is a rare life threatening cause of hematuria. The increasing frequency is attributed to increasing use of ureteral stents. A 68-year-old female presented with gross hematuria. She had prior low anterior resection for rectal cancer and a retained ureteral stent. CT abdomen and pelvis showed a large recurrent pelvic mass and a retained stent. The patient underwent cystoscopy which showed a normal bladder. Upon removal of the stent, brisk bleeding was noted coming from the ureteral orifice. Antegrade pyelogram was done which revealed a UIAF. Angiography was done and a covered stent was placed. Multiple treatment options are available. All must consider management of the arterial and ureteral side. The arterial side may be addressed by primary open repair, embolization with extra-anatomic vascular reconstruction, or endovascular stenting. The ureter can be managed with nephroureterectomy, ureteral reconstruction, placement of a nephrostomy tube, or ureteral stenting. Being minimally invasive, we believe that endovascular stenting should be the preferred therapeutic option as it also corrects the source of bleeding while preserving distal blood flow.

## 1. Introduction

Ureteroarterial fistula is a rare cause of gross hematuria [1]. With relatively few cases described in the literature, experience on management is limited to case reports and single institution case series. Krambeck et al. previously reported their experience and highlighted the difficulties in specifically diagnosing suspected ureteroarterial fistula and proposed a treatment algorithm [2]. We present an interesting case of ureteroarterial fistula and share some diagnostic and management recommendations to help localize and manage these rare entities.

## 2. Case Report

**Figure 1:**
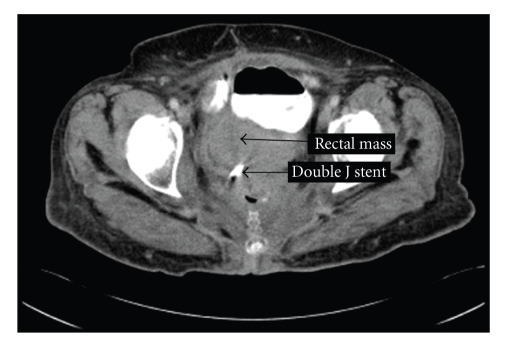
CT scan of pelvis showing rectal mass and double J stent.

A 68-year-old female presented to the emergency room with complaint of gross hematuria, intermittent right-sided flank pain, and forgotten right ureteral double J stent. A CT scan showed an enhancing 7 cm solid pelvic mass displacing the bladder and rectum (Figure 1).

She had a history of T3N0 rectal cancer status post low anterior resection with diverting colostomy followed by adjuvant chemotherapy in August 2006. Two years later, she developed pulmonary metastases. Early in 2007 she underwent right ureteral stent placement for hydronephrosis at an outside hospital. Her followup was interrupted due to her moving between two different parts of the country and between several hospitals.

She was seen at our institution in September 2007 for small bowel obstruction that required abdominal exploration, lyses of adhesions, and small bowel resection with primary anastomosis. Final pathology revealed no carcinoma. Later that year she had progression of her pulmonary metastasis and was started on additional chemotherapy. In February 2008, she developed a small right-sided extraperitoneal bladder perforation with a possible small colovesical fistula. At this time she denied any trauma, recent surgery, pneumaturia, or fecaluria. Cystoscopy and biopsy were negative. Her fistula was managed with catheter drainage alone and at 3 months a cystogram showed complete healing.

On this hospital admission at initial evaluation, given her active hematuria and pelvic mass she was taken to the operating room for cystoscopy, possible transurethral resection of tumor, control of bleeding, and possible removal of retained stent. Given the length of time the stent was indwelling, the stent was likely encrusted and would require more invasive maneuvers to remove. Given that her main problem was hematuria from an apparent pelvic mass, the likely encrusted and retained stent was a secondary objective at the time of surgery.

**Figure 2:**
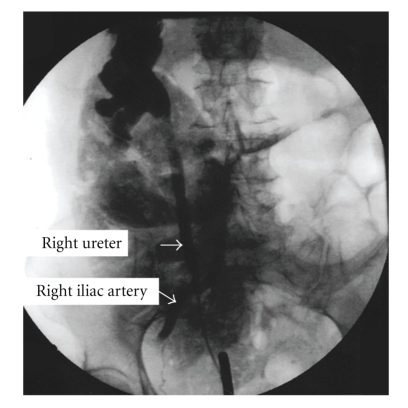
Retrograde pyelogram showing fistula between right iliac artery and right ureter. Note the cystoscope tip and ureteral catheter extending up the distal ureter.

On cystoscopic exam, her bladder showed no lesions or tumors. There was an old patch of scar on the right bladder wall consistent with the known area of bladder perforation. There was no active bleeding present. The stent was surprisingly not encrusted despite being in place for almost two years. It was removed easily with a cystoscope. Upon removal of the stent, pulsatile bleeding began from the right ureteral orifice. A retrograde pyelogram was attempted but due to the chronic indwelling stent, the ureter was patulous and contrast material would not fill the ureter but rather freely extravasate into the bladder. A guide wire was fluoroscopically advanced into the right renal collecting system and a ureteral catheter was placed into the kidney. An antegrade pyelogram showed a normal intrarenal collecting system. As the catheter was withdrawn and contrast was instilled an ureteroiliac fistula was demonstrated (Figure 2). Pulsatile arterial blood flow could now be seen coming through the ureteral catheter through the cystoscope.

Intraoperative vascular surgery and interventional radiology consultation were obtained. Given the history of prior pelvic radiation, colovesical fistula, and presumed recurrent rectal cancer in the pelvis, an open reconstructive procedure was not advised. The decision was made to proceed with placement of an intravascular stent. In order to aid in the proper positioning of the stent, an open-ended ureteral catheter was placed back into the right ureter up into the kidney.

**Figure 3:**
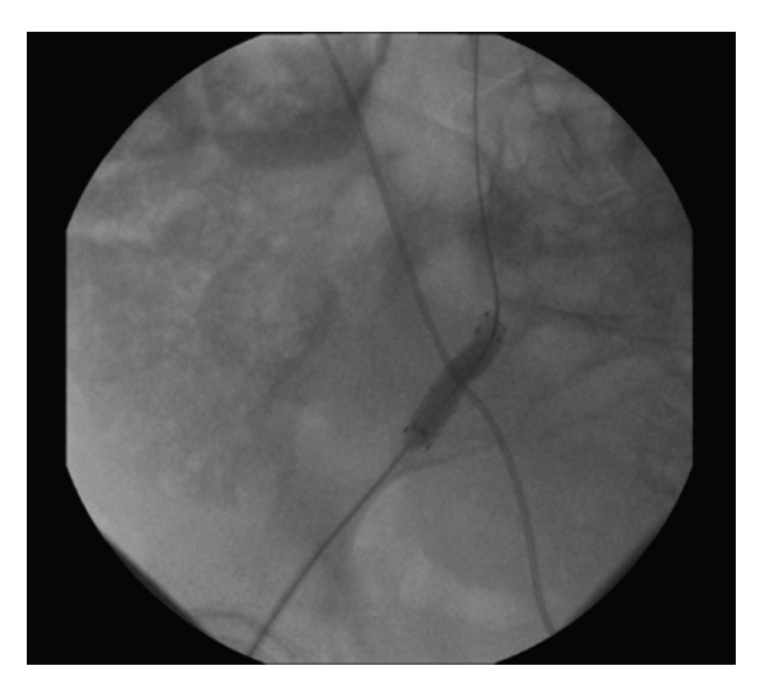
Vascular stent spanning the intersection of right iliac artery and ureter.

The patient was taken to the interventional radiology suite. Through a right common femoral access an angiogram failed to confirm or localize the fistula. Using the ureteral catheter as a guide to localize the ureter, an 8 mm × 40 mm covered vascular stent was deployed spanning the intersection of the artery and ureter (Figure 3). As a complication of the stenting the distal common femoral artery became thrombosed as evidenced on poststent angiogram. The patient was taken emergently back to the operating room where femoral thrombectomy was performed.

Postoperatively the patient had a percutaneous nephrostomy tube placed and she recovered with no further evidence of bleeding. At 2 months followup, an antegrade nephrostogram showed complete occlusion of the distal ureter at the prior fistula site. She is currently undergoing evaluation for additional chemotherapy for her metastatic and locally advanced rectal cancer.

## 3. Discussion

Although an overall rare cause of gross hematuria, ureteroiliac fistulas appear to be increasing with more frequency [3]. This is likely due to trends with more aggressive surgical resection of pelvic malignancy along with pelvic radiotherapy coupled with the liberal use of indwelling ureteral stents. The occurrence of these types of fistulas is associated with several conditions including prior pelvic surgery, pelvic radiation, chronic inflammatory conditions of the pelvis, indwelling ureteral catheters, and primary vascular disease. The presence of prior pelvic surgery, pelvic radiation, and indwelling ureteral catheters appears to be the most highly associated predisposing conditions [2, 4, 5].

In 1908, Moschcowitz reported the first case, which occurred after open bilateral ureterolithotomies and was subsequently treated with external iliac artery ligation [6].

These fistulas may form once fibrosis from prior surgery or radiation cause fixation of the ureter onto the artery where the two intersect and cross. Prior surgery or radiation may further lead to microvascular changes of the arterial vasa vasorum leading to ischemic changes. These arteries are then more susceptible to rupture, erosion, and necrosis [7]. If a ureteral catheter is present, it can act essentially as a backboard onto which a pulsatile artery will repeatedly transmit a systolic pressure wave onto the interposing arterial and ureteral wall. Ureteral catheter changes can further promote the process by causing microabrasion to the ureteral lumen. Over time a fistula can result secondary to pressure necrosis.

This theory is supported by the finding of greater degrees of arterial injury with increasing dosages of radiation which would cause greater microvascular damage [8] and that very few ureteroarterial fistulas were reported prior to the development of indwelling ureteral catheters in 1978 [9].

Ureteroarterial fistulas when they occur present with gross hematuria along with flank pain in about half the cases due to obstructing ureteral blood clots [10, 11]. Bleeding can be massive and dramatic or can be intermittent in nature. Exchange of ureteral stents can be a precipitating event provoking hematuria.

Fistulas when they occur most commonly involve the ipsilateral common iliac artery or even the external or internal iliac artery [12–14]. The site of the fistulous communication appears to occur wherever the ureter happens to cross the main arterial vessel.

A high index of suspicious is needed in order to make the diagnosis not only because the hematuria may be intermittent in nature, but also because demonstrating the diagnosis can be challenging. There is no optimal diagnostic modality as none are particularly sensitive, thus a negative result on any study does not exclude the diagnosis of ureteroarterial fistula.

CT scanning although commonly performed first in the emergency room has low sensitivity being diagnostic in 50% in one series [2]. This is partly due to the fact that fistulas are often times small and high density ureteral catheters cause significant interference in local cross-sectional imaging. The presence of pseudoaneurysm however can be a valuable clue that pathology is present. Other intraabdominal pathology such as fluid collections, masses, and hydronephrosis can easily be identified and may factor into patient management.

Cystoscopy may reveal lateralizing blood flow that may be pulsatile [1]. Performing a retrograde pyelogram is often nondiagnostic with a sensitivity of 45–60% [3, 15]. The presence of a chronic indwelling stent results in ureteral dilation and a patulous ureteral orifice. Injection of contrast rather than distending and opacifying the ureteral anatomy often times spills out of the ureter into the bladder. Identification of a fistulous communication is also difficult unless there is active bleeding as blood clots may occlude the fistulous tract. Manipulating an indwelling ureteral catheter as a provocative maneuver can induce bleeding thus facilitating the diagnosis, although the inherent pressure gradient would favor flow from the arterial system into the ureter and not vice versa [15]. Ureteroscopy with high pressure irrigation can be used but probably should be avoided as it may not be sensitive and can potentially traumatize the fistula site and result in worsening bleeding.

Angiography is even less sensitive (23–41%) [2] but the use of provocative angiography where the indwelling ureteral stent is manipulated and angiographic catheters are manipulated and infused directly over the suspected fistula site until bleeding is seen [16] has improved on the sensitivity up to 100% in one series [17].

In the cystoscopy suite, to improve diagnostic sensitivity we found that placing a ureteral catheter over a guide wire into the kidney and performing retrograde instillation of contrast as the ureteral catheter is withdrawn to be helpful—a sort of an antegrade nephrostogram using a retrograde access. It was effective in adequately distending and opacifying the ureteral anatomy and avoided the problem of distal instillation with immediate spillage of contrast into the bladder. Additionally as the catheter was withdrawn over the iliac artery by injecting contrast locally, we were able to reverse the inherent pressure gradient of the arterial system to the ureter and able to demonstrate the fistula even in the absence of active bleeding. Examination of the retrograde image in fact shows there is positive pressure instillation of contrast and preferential flow of contrast in a retrograde manner into the larger caliber distal aorta over the smaller diameter arterial system of the pelvis and lower extremity. The image is obtained under diastole when intra-arterial pressure is relatively low. Under systole there was prompt antegrade washout of contrast from the arterial system.

Once the fistula is identified we recommend placing and leaving an open-ended ureteral catheter as the immediate management options for the fistula are considered.

There are multiple treatment options available. All must consider management of the arterial and ureteral side as well as the individual patients overall clinical status. Immediate goals of management are optimization of hemodynamic status and control of bleeding if severe. Patients in shock may require immediate embolization with delayed reconstruction considered only after stabilization. Even though open reconstruction has yielded excellent results [2], it may be best to avoid an open operation in some patients with other local pathology or comorbidities that may jeopardize a reconstructive procedure. Any prior radiation treatments will further make reconstructive procedures less appealing.

In general, the arterial side may be addressed by primary open repair, open ligation, or embolization with extra-anatomic vascular reconstruction, or endovascular stenting.

The ureter can be managed with nephroureterectomy, ureteral resection with primary repair, placement of a nephrostomy tube, or ureteral stenting. Endovascular stenting has now become an appealing option over open surgical reconstruction as it is minimally invasive which allows earlier patient recovery and it appears to be as effective in controlling bleeding [18, 19]. Additionally, by maintaining distal arterial flow a second procedure to revascularize the leg is avoided. Given all this we believe that endovascular stenting should be the preferred therapeutic option.

We believe leaving an indwelling ureteral catheter is useful with respect to several management options. In the case of open reconstruction, a ureteral catheter can help in early identification of the ureter in an often times fibrotic surgical field with poor tissue planes. A common problem if endovascular stenting is chosen is failure to identify the fistula site on angiogram. The dilemma then arises as to where to deploy the stent. If a ureteral catheter is in place, one can have a landmark and confidently deploy the stent to span the ureteral catheter. Additionally the ureteral catheter can be used to aid in the placement of a nephrostomy tube so that urine can be diverted away from the exposed endovascular stent, minimizing risk of vascular stent infection.

The development of distal arterial thrombosis after vascular stenting is a known complication of arterial vascular access. This can be caused by embolization of atherosclerotic elements, propagation of clot from local intimal injury, or even arterial vascular dissection. Although a rare complication, endovascular techniques can be used to reverse the arterial occlusion [20].

## 4. Conclusion

Although rare, the incidence of ureteroarterial fistulas may be increasing. The prompt accurate diagnosis of these entities remains a challenge. We present an interesting case of ureteroiliac fistula along with few recommendations on how to improve diagnostic sensitivity during cystoscopic retrograde contrast urography. Treatment with endovascular stenting appears to be a minimally invasive, safe, and effective option.
